# *In Vitro* and Randomized, Double‐Blind, Placebo‐Controlled Trial to Determine the Efficacy and Safety of Nine Antiacne Medicinal Plants

**DOI:** 10.1155/2020/3231413

**Published:** 2020-07-09

**Authors:** Omar Said, Iyad Khamaysi, Abdalsalam Kmail, Stephen Fulder, Basheer AboFarekh, Riyad Amin, Jamila Daraghmeh, Bashar Saad

**Affiliations:** ^1^Independent Researcher, Beleaf Pharma, P.O. Box 2205, Kfar Kana, Galilee 16930, Israel; ^2^Faculty of Medicine, Technion-ITT, Rambam Medical Center, Haifa, Israel; ^3^Faculty of Arts and Sciences, Arab American University Jenin, P.O. Box 240, Jenin, State of Palestine; ^4^Qasemi Research Center-Al-Qasemi Academy, P.O. Box 124, Baqa El-Gharbia 30100, Israel

## Abstract

The present *in vitro* and randomized, double‐blind, placebo‐controlled trial aims to determine the efficacy and safety of nine Mediterranean antiacne medicinal plants. The antimicrobial, antisebum, and anti-inflammatory activities of the plant extracts were evaluated in cells from the immortalized human keratinocytes (HaCaT) and human monocytic cell line (THP-1) as well as in a double-blind, randomized, and placebo‐controlled trial. Most of the extracts showed no significant cytotoxic effects on HaCaT cells up to 250 *μ*g/ml. *Inula helenium* (IH) and *Saponaria officinalis* (SO) inhibited sebum production at 90 *μ*g/ml and 30 *μ*g/ml, respectively. The inhibition effect of SO on the growth of *Cutibacterium acnes* was 1.2 times higher than that of chloramphenicol. IH and SO extracts significantly inhibited the lipopolysaccharide- (LPS-) induced IL-6 and TNF-*α* production in THP-1 cells reaching the control levels of untreated cells at a concentration of 250 *μ*g/ml. SO, IH, and *Solanum nigrum* (SN) extracts inhibited the nitric oxide (NO) production in a dose-dependent manner. Based on these results, an antiacne herbal cream (AHC) was prepared from different portions of extracts from SO, IH, and SN, and its efficacy was evaluated in a double-blind, randomized, and controlled efficacy study with 41 acne patients, ages 18–24, who were asked to apply AHC (*n* = 27) or a placebo (*n* = 14) two to three times daily for six weeks. Results obtained indicate that the AHC has unique synergistic effects that halt sebum production, combined with highly antiseptic and anti-inflammatory activity, in which 54.95% (*t* = 19.37 *P* < 0.001) of acne inflammatory and noninflammatory lesions disappeared after two weeks, 85.3%, after five weeks (*t* = 14.19 *P* < 0.001), and 91.4%, at the end of the sixth week of application (*t* = 5.7 *P* < 0.001). In conclusion, SO, IH, and SN as single extracts and in combination as AHC showed significant antimicrobial, antisebum, and anti-inflammatory activities *in vitro* and in a double-blind, randomized, and controlled antiacne efficacy. Therefore, AHC represents an interesting alternative treatment for acne.

## 1. Introduction

Acne, also known as acne vulgaris, is a chronic inflammatory disease, predominantly affecting teenagers and young adults. The currently used acne treatments target its pathogenetic mechanisms. These include keratinocyte hyperproliferation, seborrhea, colonization of follicular ducts by *Cutibacterium acnes* (*C. acnes*), and local inflammation [1–3]. Depending on the extent and severity of the lesions, therapies vary from topical applications of antibacterial, comedolytic, and sebostatic medications, to systemic therapies with antibiotics, antiandrogenic hormones (estrogens), and retinoids [3]. These therapies are associated with significant side effects and thus could not be justified as a matter of course.

The immune system is chiefly responsible for coordinating and regulating the disease progression and even remission. New theories regarding acne development provide specific and rational directions for acne therapies. For example, *C. acnes* may be controlled by modulating specific aspects of the immune system [1, 3–6], and inhibition of 5*α*R-I or ∆^5^-3*β*-HSD by drugs can specifically target sebaceous gland function [7]. Such approaches, however, do not allow for the free use of physiological agents in the treatment of acne, as these approaches have multiple effects and other target organs [8]. Since new synthetic drugs are mostly mimics of physiological agents, they suffer from the same drawbacks, and they require long and expensive approval procedures. An attractive alternative for novel therapeutics is therefore phyototherapy.

Currently, despite the great progress in clinical medicine and pharmacology, herbal-based medicines find widespread use in most developed and developing countries. Their popularity has increased worldwide in the past five decades, probably because of the assumption that these preparations are safe and have very low levels of side effects. In the past few decades, researchers all over the world have increased their research activity on herbal-based secondary products with the aim of drug discovery. Phytochemical-based medicines are thought to act via synergistic effects (the combined effect of phytochemicals is greater than the sum of their separate effects) with reduced side effects. It can be considered a natural “straight” strategy which has evolved by nature to obtain more efficacy at low cost. In this regard, synergistic effects may be observed in the interaction between herbal-based medicines and conventional drugs or other natural products. It is important to identify and exploit these interactions since any improvement brought by such a kind of interaction can be advantageously used to achieve the best outcomes, including providing a greater benefit to patients or avoiding adverse side effects [8]. Greco-Arab herbal medicine has achieved a remarkable progress in the field of dermatology and presented tens of effective plants for treating acne and other skin diseases. For example, a large screening conducted by our group revealed that 29 medicinal plants are still used in the treatment of acne in Israel and Palestine [9]. In the present study, nine medicinal plants *Cappris spinose* (CS), *Solanum nigrum* (SN), *Ferula hermonis* (FH), *Eruca sativa* (ES), *Hypericum triquetrifolium* (HT), *Inula helenium* (IH), *Linum pubescens* (LP), *Urtica dioica* (UD), and roots of *Saponaria officinalis* (SO) or seeds of *Nigella sativa* (NS), which have been used extensively in Greco-Arab traditional medicine [7–12], were examined for their antiacne effects by testing their ability to inhibit the growth of *C. acnes,* release of proinflammatory cytokines, and excessive production of sebum. Results obtained here indicate that SO, IH, and SN were the most safe and effective plant extracts in *in vitro* tests using cells from HaCaT and THP-1 cell lines as well in *C. acnes* tests. Therefore, they were chosen for building up a cream product prototype called AHC, which was tested clinically on 21 acne patients. AHC showed unique synergistic effects; it significantly inhibited sebum production from sebaceous glands and showed antiseptic and anti-inflammatory activities. Sixty percent of acne inflammatory and noninflammatory lesions almost disappeared after five weeks of application with 75% efficacy at the end of the sixth week.

## 2. Materials and Methods

### 2.1. Preparation of Plant Extracts

Separate extracts from *Cappris spinose* (CS), *Solanum nigrum* (SN), *Ferula hermonis* (FH), *Eruca sativa* (ES), *Hypericum triquetrifolium* (HT), *Inula helenium* (IH), *Linum pubescens* (LP), *Urtica dioica* (UD), roots of *Saponaria officinalis* (SO), and seeds of *Nigella sativa* (NS) were prepared by adding 100 g of air-dried powder of each plant to one liter of 50% ethanol in distilled water and boiled for 10 minutes. The extracts were filtered through a filter paper and frozen at −70°C until use in the following experiments. The plants were collected from the Galilee region and Hermon Mountain during the periods of March and August. They were authenticated by Prof. Hasan Azaizeh (Department of Environmental Sciences, Tel-Hai Academic College, Israel) and by Dr. Omar Said (Director of Al-Maissam, a site of the Galilee Society R&D Regional Center, promoting preservation and propagation of endemic plant species used by folk healers and/or classical Arabic medicine in Israel).

### 2.2. Cell Culture

The human monocytic cell line THP-1 (ATCC 202-TIB) was obtained from ATCC (American Type Culture Collection, Manassas, VA, USA). These cells express various receptors that are found in normal monocytes and have been used as a model system for inflammatory diseases since four decades. THP-1 cells were grown in Dulbecco's modified Eagle's medium (DMEM) with a high glucose content (4.5 g/l), supplemented with 10% vol/vol inactivated fetal calf serum, 1% nonessential amino acids, 1% glutamine, 100 U/ml penicillin, and 10 *µ*g/ml streptomycin. THP-1 cells were seeded in 24-well plates at a cell density of 2 × 10^5^ cell/ml and activated with phorbol-12-myristate-13-acetate (PMA, purchased from Sigma-Aldrich, USA) (100 ng/ml) and vitamin D3 (purchased from Sigma-Aldrich, USA) (0.1 *μ*M) for 24 hours. Then, they were treated with the plant extracts (0–250 *μ*g/ml) in a fresh serum-free medium in both the absence and presence of 5 *μ*g/ml of LPS.

The immortalized human keratinocytes cell line HaCaT (obtained from the Skin Biochemistry Research Laboratory, Life Science Institute, Hebrew University, Jerusalem) were maintained in 10% Dulbecco's modified Eagle medium (Gibco Life Technologies, USA) containing penicillin (100 U/ml), streptomycin (100 mg/ml), and 10% fetal calf serum (FCS) at 37°C in a humidified atmosphere containing 5% CO_2_.

### 2.3. MTT Assay

The cell viability of the cells was determined with the MTT assay as described [13]. In brief, HaCaT cells were seeded at 2 × 103 cells/100 *μ*l culture media/well of 96-microtiter plates. Twenty-four hours after cell seeding, cells were incubated with 0–500 *μ*g of plant extract/ml of culture media for an additional 24 hours at 37°C. Following the removal of the culture media, cells were washed with phosphate buffered saline. The cells were then incubated in serum-free culture media to which 0.5 mg MTT/ml (3-(4,5-dimethylthiazol-2-yl)-2,5-diphenyltetrazolium bromide) (purchased from Sigma-Aldrich, USA) was added to each well (100 *µ*l) and incubated for a further four hours. Then, the medium was removed and the cells were incubated with 100 *µ*l of acidic isopropanol (0.08 N HCl) for 15 minutes to dissolve the formazan crystals. The absorbance of the MTT formazan was determined at 570 nm in an ELISA reader. Viability was defined as the ratio (expressed as a percentage) of absorbance of treated cells to untreated cells.

### 2.4. Lactate Dehydrogenase

Lactate dehydrogenase (LDH) activity was measured in both the cell culture media and cell lysate fractions using CytoTox 96 (cytotoxicity assay kit, Promega), in accordance with the manufacturer's instructions. The absorbance was measured at 490 nm with a 96-well microtiter plate reader (Anthos, Biochrom, Cambridge, UK) [13]. In brief, 2 × 103 HaCaT cells were seeded per well of 96-microtiter plates. Twenty-four hours after cell seeding, cells were treated with 0–500 *μ*g/ml of plant extracts. After 24 h of treatment, the culture media were collected from each well. Cell monolayers were then lysed with a cell lysis solution for 30 minutes at room temperature. LDH activity was measured in both the supernatants and cell lysate fractions. The absorbance was determined at 490 nm with a 96-well plate ELISA reader.

### 2.5. Nitrite Determination

THP-1 cells were seeded in six-well plates at a density of 2.5 × 105 cell/ml. THP-1 cells were differentiated to macrophages with PMA (100 ng/ml) and vitamin D3 (0.1 *μ*M). Twenty-four hours after cell differentiation, cells were treated for 72 h with 0–250 *μ*g/ml of IH and SO extract in a fresh serum-free medium in both the absence and presence of LPS (1 *μ*g/ml). Nitrite determinations were done in 50 *µ*l aliquots of sample mixed with 200 *µ*l of the Griess reagent [14, 15].

### 2.6. Immunoassay for Cytokines

THP-1 cells were seeded in six-well plates at a density of 2.5 × 105 cell/ml. THP-1 cells were differentiated to macrophages with PMA (100 ng/ml) and vitamin D3 (0.1 *μ*M). Twenty-four hours after cell differentiation, cells were exposed to increasing concentrations (0–250 *μ*g/ml) of IH and SO extract in a fresh serum-free medium in both the absence and presence of LPS (1 *μ*g/ml). Commercial enzyme-linked immunosorbent assay (ELISA) kits (R&D Systems, Minneapolis, MN, USA) were used to quantify the secretion of TNF-*α* and IL-6. The absorbance at 450 nm was read by a microplate reader (model 680; Bio-Rad Laboratories, Mississauga, ON, Canada) with the wavelength correction set at 550 nm. To calculate the concentration of TNF-*α* and IL-6, a standard curve was constructed using serial dilutions of cytokine standards provided with the kit.

### 2.7. Determination of Sebum Production in Sebaceous Glands Organ Culture

Skin samples (usually from breast reduction or other cosmetic surgeries) with hair (usually vellus) were obtained from Professor Yoram Milner, Head of Meyer's Skin Biochemistry Research Laboratory, Life Science Institute, Hebrew University, Givat Ram. The sebaceous glands were extracted under sterile conditions either individually or by the “shearing” method [16]. For each substance/extract tested and for isotretinoin (positive control), glands (10–20 glands/well) were floated on a preweighed nitrocellulose paper in a six-well plate [16]. The glands were distributed into the wells of the plate by rotation as they were extracted. The nitrocellulose paper with the glands was then weighed to obtain the wet weight of the glands. The glands were returned to the plate which was then incubated with growth media for primary cell cultures (F12-DMEM plus growth factors [17] and 0.02% v/v antibiotic solution (penicillin 10,000 U/ml, streptomycin 10 mg/ml, and amphotericin B 0.025 mg/ml, Biological Industries, Israel) in a tissue culture incubator (5% CO_2_, 37°C). After a recovery period of about 12 hr [18], the growth media were replaced with serially diluted, sterile, filtered preincubation media (growth media + test substance) in duplicates to give 50, 5, and 0.5 mg/ml test substance/duplicate. In each experiment, a plate containing glands with a positive control (1 mM isotretionoin in DMSO [16]) and a negative control (the solvent vehicle) was used. At the end of a 12–24 hr preincubation period, the glands were weighed again. A reduction in weight represent an indication of overall cellular damage to the glands [16]. The media in each well were then replaced with 1 ml incubation media (preincubation media + 2 *μ*Ci/ml [^14^C]-Na-acetate, 100 *μ*Ci/ml, 50 mCi/mmol in ethanol vehicle) to a final concentration of 2 *μ*M acetate and the glands were incubated for an additional 8–24 hr. After the incubation, the glands were washed thoroughly (about 10 times) with nonradioactive 0.2 mM Na-acetate in PBS, and their lipid contents were extracted and separated by the Bligh and Dyer method [19]. The lipids were dried in a speedvac to the extent they can be possibly weighed (with 0.1 mg accuracy). The dried and extracted lipids were resuspended in a known amount of resuspension buffer (chloroform : methanol 1 : 2) [18]. A part of the resuspended lipids (20–50%) were counted in a scintillation counter, and the rest were stored at −20°C.

### 2.8. Antimicrobial Activity Screening Methods

The disk diffusion experiments were carried out as described in [10]. *Cutibacterium acnes* (formerly, *Propionibacterium acnes*) (from The American Type Culture Collection, ATCC 6921) were incubated in liquid reinforced clostridium medium (RCM) for 48 h under anaerobic conditions and adjusted to yield approximately 1.0 × 108 CFU/ml. A 1.0 × 108 CFU inoculum was spread on blood agar (with sheep blood 5 to 7%) (BASB) medium plates. Discs of 6 mm diameter were prepared from Whatman no.1 filter paper, placed in a glass Petri dishes, and autoclaved for 15 min. Extract and chloramphenicol (positive control) were added to each sterile disc (25 *μ*l/disc), and the discs were dried under a laminar flow sterile bench. The final content of each disc was 5 mg of plant extracts. A sterile paper disc impregnated with test material was placed on the agar. Plates were then incubated at 37°C for 72 h under anaerobic conditions (in Gas Pak jars). 0.1 ml of the prepared bacterial broth culture was luted with 9.9 ml sterile saline.

### 2.9. Preparation of AHC Cream

The polyherbal cream “AHC” was prepared from different portions of extracts from SO, IH, SN, glycerol, and basic creams (Table 1).

### 2.10. Double-Blind, Randomized, Controlled with Placebo, Safety/Efficacy Study

Efficacy of AHC was evaluated in a double-blind, randomized, and controlled efficacy study with 41 facial acne vulgaris patients, ages 18–24, who were asked to apply AHC (*n* = 27) or placebo (*n* = 14) on the affected area, two to three times daily for a period of six weeks. The baseline assessment included personal data, description of symptoms, and details of past medical history. All patients underwent a clinical examination, and a thorough skin examination was done for the presence of black and white heads, inflamed papules and pustules, or nodules. All patients were followed up for a period of six weeks, and at each weekly follow-up visit, the improvement in the acne lesions was evaluated using the following acne severity index (ASI) in which noninflammatory count (NIC) (comedone) and inflammatory count (IC) (papule, pustule, and nodule) were determined in intervals of two, five, and six weeks. ASI values were calculated as follows:

(1) $$\begin{array}{c} \text{ASI}=\text{papules}+\left(2\times \text{pustules},\text{nodules}\right)+\left(\text{0}.25\times \text{comedones}\right)\text{.} \end{array}$$

At the end of the sixth week, the overall performance of the “AHC” cream was evaluated. Both physicians and patients were blinded to the type of the treatments, test, or placebo.

The protocol of the study was reviewed and approved (ethical approval number: AC0411, 2011) by the ethics committee of The Nazareth Hospital, E.M.M.S, Israel.

### 2.11. Statistical Analysis

Error limits cited and error bars plotted represent simple standard deviations of the mean. Usually, numerical results are given only to accuracy sufficient to specify the least significant digit. When comparing the different samples, results were considered to be statistically different when *P* < 0.05 (Student's *t*-test for unpaired samples).

## 3. Results and Discussion

The western region of the Mediterranean is covered with at least 2,600 plant species of which 700 are noted in medieval medical books for their use as medicinal herbs. According to recent ethnopharmacological studies, more than 450 of these medicinal plants are currently employed in the treatment and prevention of human diseases within the Mediterranean countries. Some of these plant species have been investigated and their bioactive ingredients extracted to treat various human diseases [20–22]. In this study, we evaluated the antiacne effects of nine medicinal plants from the western region of the Mediterranean where large portions of the population rely on them for treatment of various skin diseases [12]. The majority of the plants evaluated in this study are known to have antimicrobial activity against different microorganisms and anti-inflammatory effect [21]. However, few studies, if any, have evaluated their synergistic anti-inflammatory, antisebum, and inhibitory effects against *C. acnes,* one of the known causative agents of acne vulgaris. In the first phase of this study, MTT and LDH assays were carried out on HaCaT cells in order to evaluate nontoxic concentrations of the test plant extracts to be used in the efficacy tests. In the second phase, we evaluated the antisebum and antimicrobial effects of all test extracts. Based on the results obtained in the second phase, SO and IH extracts were selected and their anti-inflammatory effects were measured on THP-1 cell line. Based on the results obtained, a polyherbal cream “AHC” was prepared from different portions of extracts from SO, IH, and SN as described in Table 1. The efficacy of AHC cream was evaluated in an open label clinical study.

### 3.1. Cytotoxicity Measurements

MTT and LDH assays were carried out in order to evaluate nontoxic concentrations of the test plant extracts to be used in the efficacy tests. The immortalized human keratinocytes cell line, HaCaT, were used. Cells from this cell line are generally accepted as a model of skin keratinocytes [17].

**Figure 1:**
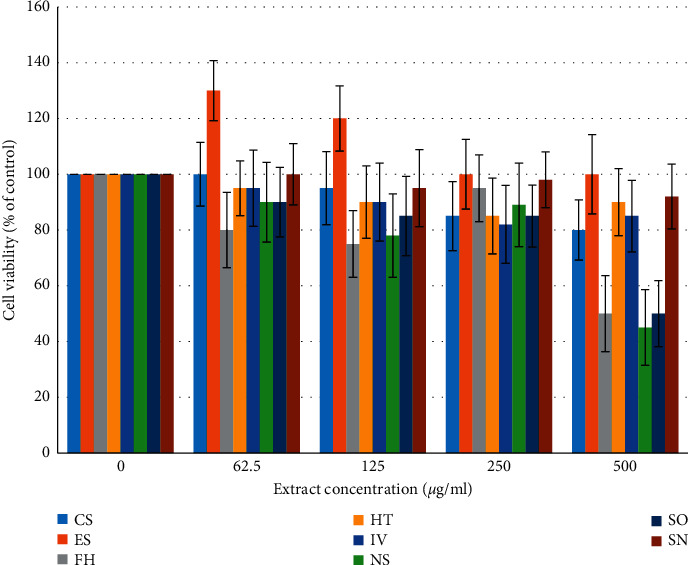
MTT assay in HaCaT cells after treatment with plant extracts (0–500 *μ*g/ml) for 24 h. *Cappris spinose* (CS), *Eruca sativa* (ES), *Ferula hermonis* (FH), *Hypericum triquetrifolium* (HT), *Inula viscosa* (IV), *Saponaria officinalis* (SO), *Nigella sativa* (NS), and *Solanum nigrum* (SN). Cell viability was defined as the ratio (expressed as a percentage) of absorbance of treated cells to untreated cells. Values represent means ± SD of three independent experiments carried out in triplicates.

The cellular metabolic activity can be evaluated with the MTT test which measures the activity of the mitochondrial enzyme succinate dehydrogenase. This test is widely used in the *in vitro* evaluation of the toxicity of plant extracts [13, 23]. We applied the MTT test to evaluate the safety of extracts from the nine test plants in cells from the HaCaT. Cells were exposed to increasing concentrations (1–500 *μ*g/ml of culture medium) of extracts for 24 h. No sign of any negative effects were observed after treatment with concentrations up to 250 *μ*g/ml (Figure 1). Concentrations higher than 250 *μ*g/ml caused a significant reduction in the cell viability.

Plasma membrane integrity can be evaluated by measuring the extracellular lactate dehydrogenase activity. Lactate dehydrogenase, an enzyme located in the cytoplasm, catalyzes the conversion of lactate and pyruvate. When lactate dehydrogenase is found within the media on the cells, there are two possible causes: the first is cellular death and the second is a “leak” in a cell membrane [13]. When cells are disrupted, the lactate dehydrogenase activity is elevated. The results obtained indicate no significant changes of the LDH levels in the culture medium after exposure to plant extracts at a concentration up to 125 *μ*g/ml. A slight, but not significant, increase was observed after treatment with concentrations of 250 and 500 *μ*g/ml (data not shown). Based on the MTT and LDH results, concentrations below 250 *μ*g/ml were used in the following experiments.

### 3.2. Antibacterial Activities

**Figure 2:**
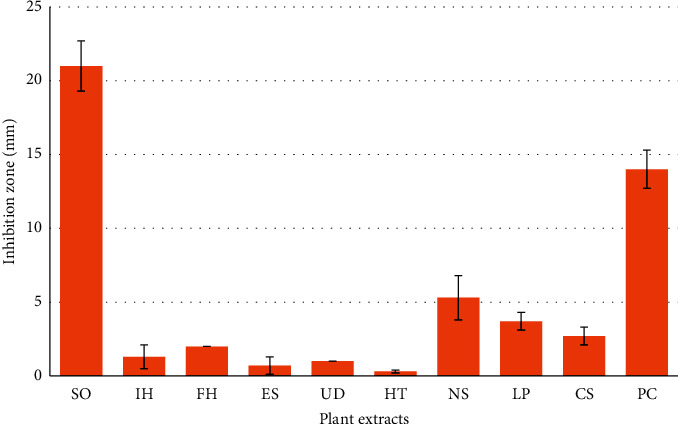
Antibacterial effects of the plant extracts. The inhibition zones' diameters were measured to evaluate the antimicrobial activities of the plant extracts against *Cutibacterium acnes*, compared with common antiacne plants (PC). *Saponaria officinalis* (SO), *Inula helenium* (IH), *Ferula hermonis* (FH), *Eruca sativa* (ES), *Urtica dioica* (UD), *Hypericum triquetrifolium* (HT), *Nigella sativa* (NS), *Linum pubescens* (LP), and *Cappris spinose* (CS). The final content of each disc was 5 mg of extract. Values represent means ± SD of three independent experiments carried out in triplicates.

**Figure 3:**
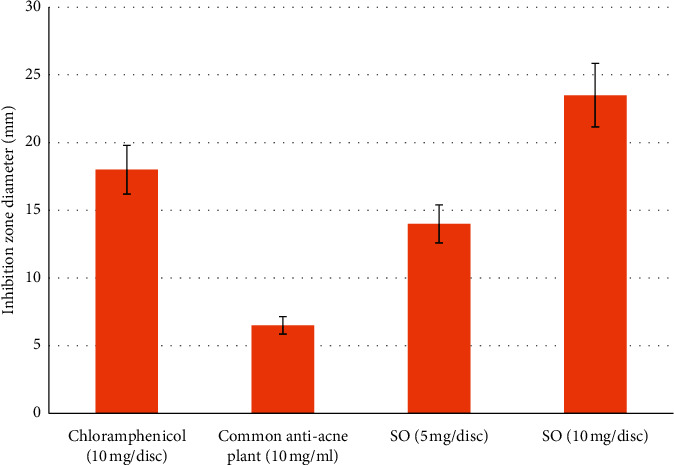
Evaluation of antibacterial effects of *Saponaria officinalis* (SO) extract and chloramphenicol, as a positive control, by measuring the inhibition zone diameters.

Plants can be efficient in the treatment of acne vulgaris because of four mechanisms including antibacterial, anti-inflammatory, antioxidant, and antiandrogen activities [23]. The plants with essential oils, flavonoids, alkaloids, and phenolic compounds are well known for their antibacterial effects including inhibitory action against acne-inducing bacteria [24–28]. *C. acnes* has been recognized as pus-forming bacteria triggering an inflammation in acne. The antimicrobial activities of nine medicinal plants against *C. acnes* were evaluated here using disc diffusion method. Inhibition zone diameters were measured to evaluate the antimicrobial activities of the plant extracts against *C. acnes*, compared with chloramphenicol and common antiacne plants. The results showed that six extracts could effectively inhibit the growth of *C. acnes*. SO showed an *in vitro* antibacterial activity against *C. acnes* 1.2 times higher than that of the reference antibiotic (chloramphenicol) (Figure 2). *Nigella sativa* showed weak activity (5.3 ± 1.5 mm) compared to that of chloramphenicol (14 ± 0.0 mm). The remaining extracts showed either very weak or no activity (0.3 ± 0.7 to 3.7 ± 0.6 mm) (Figures 2 and 3).

### 3.3. Antisebum Effects

**Figure 4:**
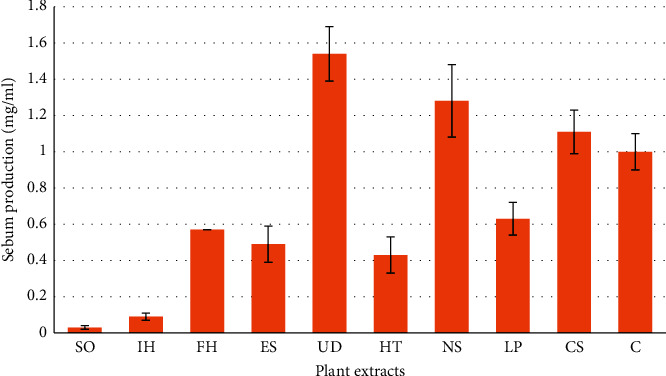
Radioactive acetate incorporation into total lipids as a function of *Cappris spinose* (CS), *Eruca sativa* (ES), *Ferula hermonis* (FH), *Hypericum triquetrifolium* (HT), *Inula helenium* (IH), *Linum pubescens* (LP), *Nigella sativa* (NS), *Saponaria officinalis* (SO), *Urtica dioica* (UD), and control cells (C)*. Sebum* production of pairs of glands is normalized relative to that of untreated glands, and the results are fitted with exponential decay curves. The bar heights represent the 50% inhibitory dosages (mg/ml) at which 50% reduction of the untreated control lipid incorporation was obtained. Values represent means ± SD of three independent experiments carried out in triplicates.

Sebum plays important physiological roles in human skin. Excess sebum production contributes to the pathogenesis of acne vulgaris, and suppression of sebum production reduces acne incidence and severity. Four different experiments were conducted in the course of this study. For comparisons between different experiments, the incorporation of radioactive acetate into extract treated glands was normalized to matched control glands. The results are summarized in Table 2. These normalized results were plotted and an exponential decay curve was fitted to the data. The point at which 50% reduction in total lipid incorporation (EC50 in Table 2) is reached was calculated for each from the equation parameters (Figure 4). Using these parameters, the 95% confidence limits for D50 were also calculated using the decay constant, t1 error (since EC50≃t1*∗*(ln 2) ± error(t1)*∗*ln(2)) (Figure 4).

The effects obtained with the positive control isotretinon were in good agreement with published results. Isotretinoin caused a 50% reduction in proliferation and ∼70% reduction in total lipids production at a concentration of 10^−5^ M [27]. The EC50 value obtained for this compound (9.4 × 10^−6^ M) is quite close to this number, which should be expected. At the same time, the SO and IH extract achieved the same effect but at a higher concentration, 0.03 ± 0.02 and 0.09 ± 0.01 mg/ml, respectively (Figure 3). The test extracts inhibited the production at relatively high concentrations (EC50 0.43–1.54 mg/ml).

### 3.4. Proinflammatory Cytokines IL-6, TNF-*α*, and NO

Acne is an inflammatory disease where several cytokines play key roles in mediating acute inflammatory responses, such as IL-1*β*, IL-6, IL-8, and TNF-*α*, which are extremely potent inflammatory molecules. *C*. *acnes* were found to stimulate monocytes to secrete proinflammatory cytokines including TNF-*α*, IL-1*β*, IL-6, and IL-8 [26]. These cytokines originate from white blood cells, keratinocytes, and sebocytes in and around the damaged follicle, as the host cross-talks with skin defense and immune cells to fight and protect against the infection [26, 29, 30]. The inhibition of the overproduction of such cytokines, especially proinflammatory cytokines (TNF-*α* and IL-6) and nitric oxide, may prevent or suppress inflammation in acne patients. Reactive oxygen species (ROS) are generated from the hypercolonization of *C. acnes* [30]. Although ROS perform a useful function in the skin barrier against acne microbes, excess formation affects skin condition by activating neutrophil infiltration. ROS including singlet oxygen, superoxide anion, hydroxyl radical, hydrogen peroxide, lipid peroxide, and nitric oxide (NO) play an important role in inflammatory acne as well as in tissue injury. ROS stimulate the formation of nuclear factor *κ*B (NF‐*κ*B), promote TNF-*α* formation, and consequently activate T lymphocytes and keratinocytes. The cytokines IL-6, TNF-*α*, and IFN, lipopolysaccharide (LPS), transforming growth factor (TGF), and prostaglandin (PG) are then produced and released [29–33].

**Figure 5:**
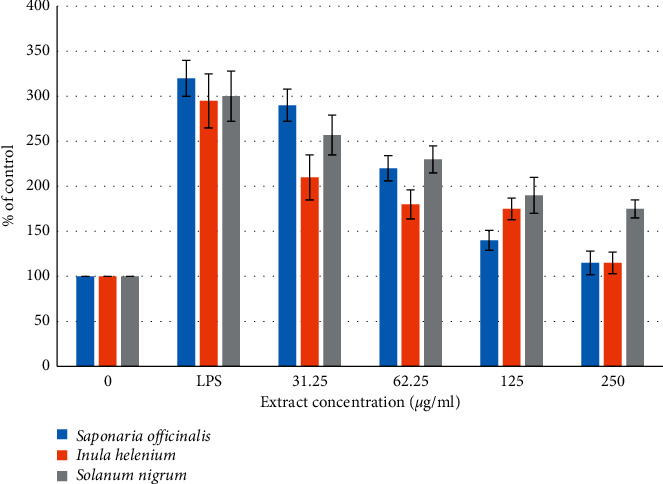
Dose-dependent inhibition of LPS-mediated production of nitric oxide (NO) in LPS-treated THP-1 cells by *Saponaria officinalis* (SO) and *Inula helenium* (IH) extracts. For each concentration treatment, the level of NO release is represented as a percentage of the control set at 100%. Values represent means ± SD (^*∗*^*P* < 0.05 significant as compared to LPS alone) of three independent experiments carried out in triplicates.

LPS-activated human monocytic cell line THP-1 were used here to measure the anti-inflammatory effects of SO and IH extracts which showed in previous experiments significant antibacterial and antisebum activities. LPS-activated human monocytic cell line THP-1 are known to produce various proinflammatory cytokines, such as TNF-*α*, IL-1*β*, and IL-6 as well as NO [15, 34–36]. The production of IL-6, TNF-*α*, and NO by cultured THP-1 was tested in the culture supernatants using commercial enzyme-linked immunosorbent assay (ELISA) kits.  Figure 5 shows the dose-dependent inhibition of the LPS-mediated production of nitric oxide (NO). SO, SN, and IH extracts inhibited the NO production by cultured THP-1 in a dose-dependent manner reaching the control levels of untreated cells at a concentration of 250 *μ*g/ml (Figure 5). It was found that THP-1 produce detectable amounts of IL-6 and TNF-*α* after stimulation with LPS. Maximal TNF-*α* and IL-6 concentrations were detectable in the culture supernatants 4 h and 6 h after LPS stimulation, respectively. Therefore, the 4 h and 6 h time points were used to characterize the effects of SO and IH extracts on TNF-*α* and IL-6 production by cultured THP-1, respectively. The anti-inflammatory activity and release of cytokines like TNF-*α* is linked with an inflammatory mediator nuclear factor-kappa B (NF-*κ*B) [26, 30]. NF-*κ*B is a transcription factor that resides in the cytoplasm of every cell and its constitutive activation is linked with *C. acnes* infection. The suppression of the cytokines TNF-*α* can possibly be due to blocking activation of common transcription factor such as NF-*κ*B involved in their induction [26, 30].

**Figure 6:**
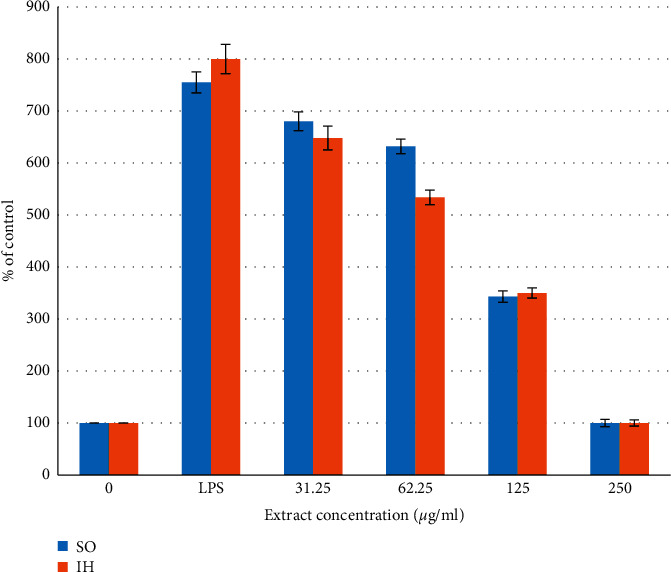
Dose-dependent inhibition of LPS-mediated production of TNF-*α*-untreated and LPS-treated THP-1 cells by *Saponaria officinalis* (SO) and *Inula helenium* (IH) extracts. For each concentration treatment, the level of TNF-*α* release is represented as a percentage of the control set at 100%. The bar heights represent the values of means ± SD (^*∗*^*P* < 0.05 significant as compared to LPS alone) of three independent ELISA experiments carried out in triplicates.

**Figure 7:**
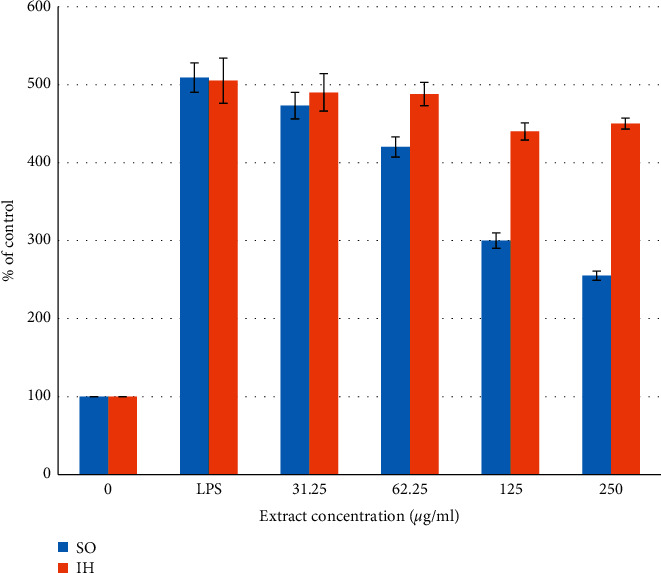
Dose-dependent inhibition of LPS-mediated production of IL-6-untreated and LPS-treated THP-1 cells by *Saponaria officinalis* (SO) and *Inula helenium* (IH) extracts. For each concentration treatment, the level of IL-6 release is represented as a percentage of the control set at 100%. The bar heights represent the values of means ± SD (^*∗*^*P* < 0.05 significant as compared to LPS alone) of three independent ELISA experiments carried out in triplicates.

Figures 6 and 7 show the TNF-*α* and IL-6 secretion into the culture supernatant of untreated and LPS-treated THP-1 cells. Both plant extracts inhibited the TNF-*α* production in a dose-dependent manner reaching the control levels of untreated cells at a concentration of 250 *μ*g/ml (Figure 6). SO inhibited the IL-6 secretion by about 50% with no effects on the production levels of IL-6 at 250 *μ*g/ml, while no effects were seen after treatment with IH (Figure 7). Saponins from SO enhanced the phagocytic, bactericidal, and adhesion activities of polymorphonuclear leukocytes. Optimal conditions of saponin treatment (dose and duration) were determined for mice. Saponins promoted the maturation of human peripheral blood dendritic cells, which was observed by high expression of CD83 (terminal differentiation marker) and CD86 (bone-stimulating molecule) and of HLA-DR and HLA-ABC molecules on the cell membrane. Saponins modulated the production of TNF-*α*, IL-1*β*, IL-4, IL-6, and IFN-*γ* in cultured peripheral blood intact cells. The results help to understand some mechanisms of the effects of saponins extracted from SO on the cellular and humoral factors of innate immunity and demonstrate good prospects of their practical use [37].

### 3.5. Double-Blind, Randomized, Controlled with Placebo, Safety/Efficacy Study of AHC

Many plants seem to have inhibitory effects on the growth of *C. acnes in vitro*. Also, some plants have been shown to have anti-inflammatory and antisebum properties [23–25, 27, 28, 38]. However, there are a few clinical evidences about the effectiveness and safety of these plants in the treatment of acne and other skin infections. As mentioned above, SO and IH showed a dose-dependent effect on the release of IL-6, TNF-*α*, and NO as well as showing significant antibacterial and antisebum effects. Since these plants have different chemical components, when having them in a mixture, strong beneficial effects may result. In addition, the combination of plants may help the body to manage potentially undesirable effects that each plant might have when used alone and when in combination/formulation they might play a curative role. It is therefore preferable to use plant combination instead of relying on a single plant [20–23]. In this regard, AHC was prepared according to their highest antimicrobial activity and antisebum and anti-inflammatory effect where they showed a significant reduction in all tested parameters.

**Figure 8:**
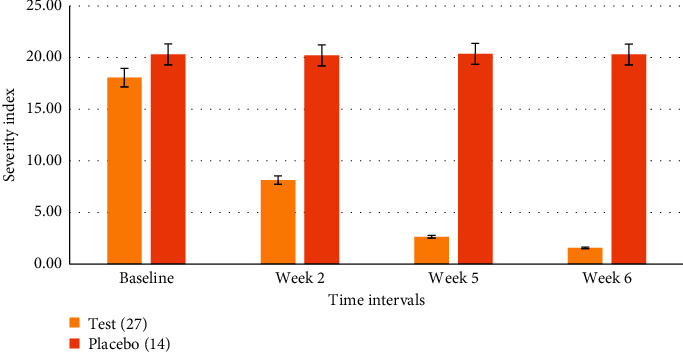
Double-blind, randomized, controlled with placebo, safety/efficacy study of antiacne herbal cream (AHC) with 41 facial acne vulgaris patients, ages 18–24, who were asked to apply AHC (*n* = 27) or placebo (*n* = 14) on the affected area, two to three times daily for a period of six weeks. Effects of AHC on inflammatory and noninflammatory severity index (ASI) compared to placebo.

Based on the initial antimicrobial, antisebum, and anti-inflammatory screening test, a polyherbal cream “AHC” was prepared from different portions of extracts from SO (roots), IH (flowers and leaves), SN (leaves), glycerol, and basic creams (Table 1). SO was shown in various *in vitro* and *in vivo* studies to exhibit antibacterial, antihistamine, and anti-inflammatory properties [39–41]. The efficacy of AHC cream was evaluated in a double-blind, randomized, and controlled study with placebo. Results obtained indicate that the AHC has unique synergistic effects that halt sebum production, combined with highly antiseptic and anti-inflammatory activity, in which 54.95% (*t* = 19.37 *P* < 0.001) of acne inflammatory and noninflammatory lesions almost disappeared after two weeks of application, 85.3%, after five weeks, (*t* = 14.19 *P* < 0.001), and 91.4%, at the end of the sixth week (*t* = 5.7 *P* < 0.001) (Table 3 and Figure 8). The three herbs are on the INCI list of approved cosmetic ingredients for sale in the EU (Table 4). There was no evidence of toxicity or adverse effects.

**Figure 9:**
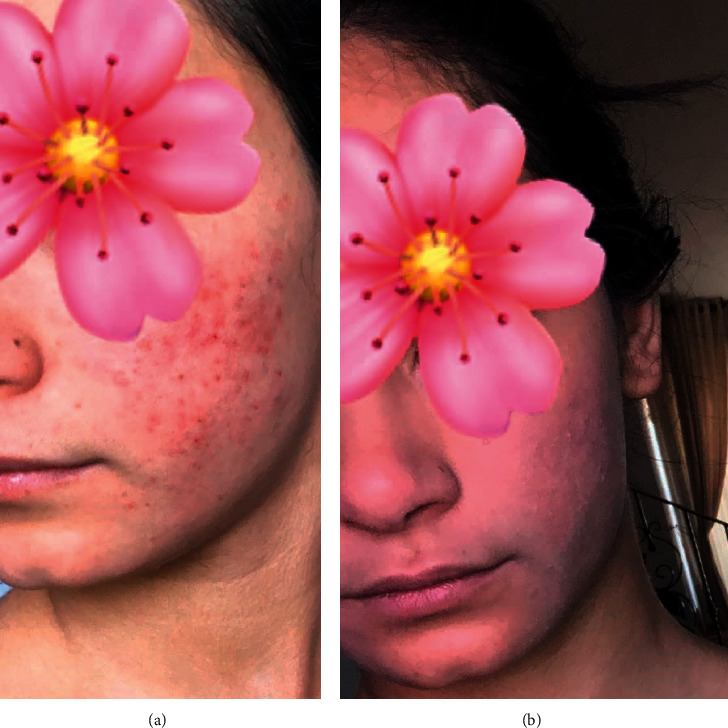
An acne patient before (a) treatment and after treatment (b) with antiacne herbal cream (AHC). The patient was asked to apply AHC on the affected area, two to three times daily for a period of one week.

There are many published studies that investigated the antiacne effects of medicinal plants and their extracts [9–12, 26–30, 32]. However, to our knowledge, this is the first study to determine the potential use of ethanolic extracts of SO, IH, and SN as antimicrobial agents against *C. acnes*. In addition, it is the first study to evaluate the anti-inflammatory effect of these extracts on the release of IL-6, TNF-*α*, and NO from cells from the THP-1 cell line. Moreover, this study shows a novel antisebum, antimicrobial, and anti-inflammatory effect when a mixture of plant extracts (AHC) was used. The combinations of the medicinal plants showed a synergic antimicrobial and anti-inflammatory activity, while a single constituent may or may not have a pharmacological effect. Topical application of AHC showed in a clinical study antiseptic and anti-inflammatory activities, in which 60% of acne inflammatory and noninflammatory lesions disappeared after five weeks of application and 75%, at the end of sixth week (Figures 8 and 9).

## 4. Conclusions

Acne vulgaris is a chronic inflammatory disorder in which *Cutibacterium acnes* plays a critical role in its development when it overgrows in the pilosebaceous unit. The present study was conducted to evaluate the antimicrobial, antisebum, and anti-inflammatory activity of nine Mediterranean plant extracts separately and in combination for the treatment of acne. This study shows a novel antimicrobial, antisebum, and anti-inflammatory effect of extracts from SO and IH when used as single. A double-blind, randomized, and controlled efficacy study was conducted with 41 facial acne vulgaris patients (ages 18–24). The patients were asked to apply AHC (*n* = 27) or placebo (*n* = 14) on the affected area, two to three times daily for a period of six weeks. This study revealed that the combinations of the SO, IH and SN showed synergistic effects which dramatically halt sebum production from sebaceous glands, combined with highly antiseptic and anti-inflammatory activity, where 54.95% of acne inflammatory and noninflammatory lesions almost disappeared after two weeks of application, 85.3%, after five weeks, and 91.4%, at the end of sixth week. These results suggest that AHC shows favorable antiacne effects through antibacterial and antisebum effects as well as through significant reduction in the number of inflammatory and noninflammatory lesions.

## Figures and Tables

**Table 1:** Plant extracts used in the preparation of the antiacne herbal cream (AHC).

Botanical name	English common name (part used)	Weight (g/50 mL)	%
*Saponaria officinalis* L.	Saponaria (roots)	0.16	0.32
*Inula helenium*	Elecampane (flowers and leaves)	0.4	0.8
*Solanum nigrum*	Black nightshade (leaves)	0.4	0.8
Glycerin		2.0	4.0
Basic cream		47.04	94.08
Total		50	100

**Table 2:** The effect of *Linum pubescens* (LP), *Nigella sativa* (NS), *Hypericum triquetrifolium* (HT), *Ferula hermonis* (FH), *Cappris spinose* (CS), *Inula helenium* (IH), *Eruca sativa* (ES), *Saponaria officinalis* (SO), *Urtica dioica* (UD), and *Solanum nigrum* (SN) extracts on sebum production in sebaceous glands organ culture was measured though assessment of acetate incorporation into sebaceous gland total lipids in the absence and presence of ethanolic extracts. Each result represents an average of nine repeated experiments.

Extracts (mg/ml)	LP	NS	HT	FH	CS	IH	ES	SO	UD
0.00	1.00	1.00	1.00	1.00	1.00	1.00	1.00	1.00	1.00
0.05	0.59	0.91							
0.06			1.00	0.71	0.89	0.50	0.81	0.18	0.67
0.11	0.87								
0.19			0.83	0.51	0.89	0.34	0.51	0.33	0.75
0.33	0.75								
0.5		0.80							
0.56			0.29	0.56	0.57	0.08	0.60	0.00	0.41
1.00	0.35								
1.67			0.18	0.23	0.47	0.00	0.08	0.00	0.66
3.00	0.13								
5.00		0.03	0.03	0.02	0.22	0.01	0.03	0.04	0.26
*r*	−0.83	−1.00	−0.96	−0.94	−0.94	−0.94	−0.94	−0.80	−0.89
*P*	0.05	0.05	0.01	0.01	0.01	0.01	0.01	0.10	0.05
#/point	5	10	5	5	2	2	2	2	2
EC50 (mg/ml)	**0.67**	**1.28**	**0.43**	**0.57**	**1.11**	**0.09**	**0.49**	**0.03**	**1.54**
STDEV	0.41	0.30	0.09	0.33	0.19	0.02	0.22	0.01	1.18

*r*: Spearman matched pair rank correlation coefficient; *P*: probability that *r* is random (one tail); #/point: number of glands used per data point; EC50: calculated concentration (mg/ml) at which 50% of the untreated control lipid incorporation is obtained; EC50: 95% confidence limits for EC50 (mg/ml) (^∗^P < 0.05 significant as compared to control).

**Table 3:** Acne severity index (ASI) was evaluated in a double-blind, randomized, and controlled efficacy study with 41 acne patients, aged 18–24 years, who were asked to apply antiacne herbal cream (AHC) (*n* = 27) two to three times daily for six weeks.

ASI (acne severity index) of AHC group				
Patient no.	Baseline	Week 2	Week 5	Week 6
	ASI	ASI	ASI	ASI
1	16.25	7.50	1.75	2.25
2	15.00	7.50	2.50	1.00
3	18.75	9.50	3.25	3.00
4	14.25	5.00	0.00	0.00
5	13.00	5.00	0.00	0.00
6	8.75	1.25	0.50	0.25
7	12.75	3.50	0.25	0.00
8	21.25	10.00	3.75	3.25
9	14.00	5.25	2.25	1.00
10	21.50	10.25	2.00	1.00
11	26.00	13.00	4.50	2.00
12	21.75	9.25	5.00	2.25
13	30.50	16.25	8.25	5.50
14	11.50	4.75	0.00	0.00
15	14.25	6.50	3.25	1.50
16	12.25	7.50	1.00	0.25
17	12.00	5.25	0.00	0.00
18	19.50	9.00	2.00	1.00
19	28.75	11.25	4.00	2.00
20	17.50	5.75	2.00	1.00
21	28.50	15.50	4.75	3.75
22	20.25	9.25	4.50	2.00
23	18.75	10.00	4.75	2.25
24	17.50	7.25	2.00	1.00
25	21.00	10.50	5.00	4.00
26	13.25	4.25	0.00	0.00
27	19.00	9.75	4.25	2.00
Average	18.06	8.14	2.65	1.56
STDEV	5.65	3.5098	2.129	1.4321
St. err.	1.23	0.77	0.46	0.31
	*t*	19.37	14.19	5.70
	*P*	<0.05	<0.05	<0.05

**Table 4:** Acne severity index (ASI) was evaluated in a double-blind, randomized, and controlled efficacy study with 41 acne patients, aged 18‐24 years, who were asked to apply placebo (n = 14) 2-3 times daily for 6 weeks.

ASI (acne severity index) of placebo group				
Patient no.	Baseline	Week 2	Week 5	Week 6
	ASI	ASI	ASI	ASI
1	13.75	13.50	13.50	13.25
2	14.00	13.75	13.75	13.50
3	23.00	22.50	22.50	22.50
4	23.50	23.25	23.25	23.25
5	10.50	11.00	11.00	11.00
6	15.00	15.25	15.25	15.50
7	16.50	16.00	16.50	16.00
8	16.50	16.25	16.25	16.25
9	20.50	20.25	20.25	20.25
10	26.25	26.00	26.50	26.25
11	32.00	32.50	33.00	33.00
12	31.50	31.25	31.25	31.25
13	26.25	26.00	26.00	26.00
14	15.25	15.50	16.00	16.25
Average	20.32	20.21	20.36	20.30
STDEV	6.86	6.83	6.88	6.90
St. err.	1.50	1.49	1.50	1.51
	*t*	1.19	−2.28	1.00
	*P*	0.250	0.038	0.33

## Data Availability

The data used to support the findings of this study are available from the corresponding author upon request.
